# Elevated IL-19 Serum Levels in Patients With Pernicious Anemia and Autoimmune Gastritis

**DOI:** 10.3389/fimmu.2022.887256

**Published:** 2022-04-11

**Authors:** Chiara Della Bella, Antonio Antico, Maria Piera Panozzo, Nagaja Capitani, Marisa Benagiano, Luisa Petrone, Annalisa Azzurri, Sara Pratesi, Sofia D’Elios, Fabio Cianchi, Diana Ortiz-Princz, Nicola Bizzaro, Mario Milco D’Elios

**Affiliations:** ^1^ Department of Experimental and Clinical Medicine, University of Florence, Florence, Italy; ^2^ Laboratory of Clinical Pathology, ULSS7 Pedemontana, Hospital Alto Vicentino, Santorso, Italy; ^3^ Department of Life Sciences, University of Siena, Siena, Italy; ^4^ Endocrinology Unit, Careggi Hospital, Florence, Italy; ^5^ Laboratory of Clinical Pathology, Toscana Centro Hospital, Firenze, Italy; ^6^ Department of Clinical and Experimental Medicine, University of Pisa, Pisa, Italy; ^7^ Laboratory of Molecular Microbiology, Autonomous Service Institute of Biomedicine “Dr. Jacinto Convit”, Caracas, Venezuela; ^8^ Laboratory of Clinical Pathology, San Antonio Hospital, Tolmezzo, Italy; ^9^ Laboratory of Clinical Pathology, Azienda Sanitaria Universitaria Integrata, Udine, Italy

**Keywords:** pernicious anemia, autoimmune gastritis, interleukin 19, serum, gastric mucosa

## Abstract

Pernicious anemia (PA) is a megaloblastic anemia consisting of hematological, gastric and immunological alterations. The immunopathogenesis of PA is sustained by both autoantibodies (e.g. intrinsic factor (IFA) antibodies and anti parietal cell (PCA) antibodies and autoreactive T cells specific for IFA and the parietal cell proton pump ATPase. Iron deficient anemia (IDA) is a microcytic anemia and represents the most common cause of anemia worldwide. Our work aimed to investigate serum levels of several interleukins (IL) of the IL-20 cytokine subfamily in patients with PA, with IDA and in healthy subjects (HC). We compared serum levels of IL-19, IL-20, IL-26, IL-28A and IL-29 in 43 patients with PA and autoimmune gastritis, in 20 patients with IDA and no autoimmune gastritis, and in 47 HC. Furthermore, we analyzed the IL-19 cytokine production by gastric lamina propria mononuclear cells (LPMC) in eight patients with PA and four HC. We found that patients with PA have significantly higher serum levels of IL-19 (163.68 ± 75.96 pg/ml) than patients with IDA (35.49 ± 40.97 pg/ml; p<0.001) and healthy subjects (55.68 ± 36.75 pg/ml; p<0.001). Gastric LPMC from all PA patients were able to produce significantly higher levels of IL-19 (420.67 ± 68.14 pg/ml) than HC (53.69 ± 10.92 pg/ml) (*p*<0.01). Altogether, our results indicate that IL-19 serum levels are significantly increased in patients with PA but not with IDA and that IL-19 is produced *in vivo* in the stomach of PA patients. These data open a new perspective on PA pathogenesis and suggest that IL-19 may represent a novel important tool for the management of patients with PA.

## Introduction

Anemia is a condition in which the haemoglobin concentration is lower than normal and might be macrocytic or microcytic. Pernicious anemia (PA) is a megaloblastic anemia consisting of hematological, gastric and immunological alterations. It is an autoimmune disease with a multifactorial etiology involving genetic susceptibility, environmental and immunological factors. Estimate of PA prevalence is 0.1% in the general population which increases to 1.9% in patients aged older than 60 years (1). In older adults of European and African ancestry it is more common (4.0% and 4.3%, respectively) than in people of Asian descent (0.43%) (2, 3).

The immunopathogenesis of pernicious anemia is sustained by autoreactive T cells and by autoantibodies, e.g. intrinsic factor antibodies (IFA) and parietal cell antibodies (PCA). Autoimmunity begins with the activation of gastric dendritic cells, which in turn trigger CD4^+^ T cell lymphocytes in perigastric lymph nodes (4, 5). These autoreactive CD4^+^ T cells as well as the autoantibodies produced by autoreactive B cells are specific for intrinsic factor and the proton pump ATPase, thus attack gastric parietal cells and ultimately lead to gastric cell death and gastric atrophy (6–8). The gastric mucosa atrophy impairs absorption of dietary cobalamin resulting in vitamin B_12_ deficiency.

Patients develop unexplained fatigue, pallor, cardio-respiratory symptoms like shortness of breath and tachycardia, gastrointestinal manifestations and neurologic abnormalities such as memory loss, poor concentration and paresthesia. It often occurs in combination with other autoimmune disorders such as autoimmune thyroiditis, Addison’s disease, vitiligo, and type 1 diabetes (9, 10).

PA diagnosis includes the serum B_12_ assay to establish cobalamin deficiency (B_12_<200ng/L with normal or reduced folate levels) and the detection of serum IFA or PCA (11).

On the other hand, iron deficiency anemia (IDA) is the most common cause of anemia worldwide affecting >12% of the world’s population, especially women, children, and individuals living in under-resourced and middle-income countries. Diagnosis of IDA is based on medical history, microcytic anemia, and a serum ferritin level <15 mg/L (12).

The interleukin (IL)-20 family members, IL-19, IL-20, IL-22, and IL-26 are grouped into the same family based on shared common receptor subunits (the IL-20 receptor β chain, IL-20Rβ) and signaling pathways. All IL-20 subfamily members activate the Janus Kinase (JAK) and signal transducer and activator of transcription (STAT) pathway, particularly STAT3 (13, 14). These subfamily members are part of the larger IL-10 family of cytokines, which besides IL-10 itself, includes the more distantly related IL-28A and IL-29 (which are also known as IFNλ2 and IFNλ1, respectively) (15). The interleukin−20 (IL−20) cytokine subfamily constitutes a key link between the immune system and epithelial tissues, and exerts essential functions in innate mucosal immunity (16). IL-28A and IL-29 are able to induce antiviral responses similar to those of type I interferons and mainly act on epithelial cells (17).

IL-19, IL-20, IL-22, IL-26, IL-28A and IL-29 are produced by different inflammatory cells, primarily keratinocytes, osteocytes, epithelial cells, myeloid cells, B and T cells (14, 18, 19). IL-19 expression can be induced by different stimuli such as TNF-α and IL-17 (20).

IL-19 functions as an anti-inflammatory and pro-angiogenic factor (21). IL-19 was discovered in 2000 and has been implicated in different diseases, such as psoriasis, type I diabetes, endotoxic shock, periodontal disease, vascular diseases and rheumatoid arthritis (22, 23). Xiao et al. recently identified IL-19 as a potent cytokine able to promote the expansion and proliferation of neutrophils. The administration of IL-19 protected wild-type mice from neutropenia induced by both chemotherapy and radiation, demonstrating the therapeutic efficacy of this cytokine. It still remains unknown whether IL-19 has additional effects on other organs or tissues but its potential application in neutropenic state is undoubted, being more efficient than granulocyte colony-stimulating factor (G-CSF). Cancer patients who underwent chemotherapy and had reduced activity to promote bone marrow neutrophil formation, had striking downregulation of serum IL-19 (24).

We wondered whether levels of IL-19 and other related cytokines could be abnormal in patients with anemia, such as PA or IDA. We thus measured serum IL-19, IL-20, IL-26, IL-28A and IL-29 levels in patients with PA, in patients with IDA and in healthy subjects (HC). We then investigated the *in vivo* production of IL-19 by lamina propria mononuclear cells (LPMC) derived from the stomach of patients with PA and controls.

## Materials and Methods

### Subjects and Samples

Upon approval of the local Ethical Committee (Ethic statement n.14936/CAM_BIO), 110 subjects were enrolled in this study. Briefly, we studied serum levels of IL-19 and other related cytokines in 43 untreated patients with PA and autoimmune gastritis (diagnosed by histology), 20 untreated patients with IDA and no autoimmune gastritis (as defined by histology), and 47 HC. Patients groups, age and sex are detailed in **Table 1**. Eight PA patients out of 43 suffered also from autoimmune thyroiditis. No other autoimmune comorbidities were present in PA, IDA and HC subjects. None of the patients suffered from peptic ulcer, gastric cancer, gastric lymphoma nor had proton pump inhibitors assumption in the previous six months. Active *H. pylori* infection, as ruled out by histopathology, was not present in any patient or control. All PA, IDA patients and controls were investigated by serology (Helori CTX, Eurospital, Trieste, Italy) and three PA patients were seropositive for *H. pylori.* Diagnosis of PA was based on medical history, macrocytic anemia in peripheral blood, erythroblastosis with megaloblastic changes in bone marrow, low serum levels of vitamin B_12_ (below 200ng/L). All participants were tested for intrinsic factor and anti-parietal cell autoantibodies. All PA patients had serum intrinsic factor autoantibodies, as assessed by immunofluorenzymatic assay (EliA, Thermo Fisher, Uppsala, Sweden) and/or anti-gastric parietal cells autoantibodies detectable by indirect immunofluorescence assay on rodent tissue (**Table 2**) (8, 11). 12 out of 43 PA patients had serum gastric parietal cells autoantibodies, 15 PA patients had serum autoantibodies against intrinsic factor, and 16 PA patients had both PCA and IFA autoantibodies. Diagnosis of IDA was based on medical history, microcytic anemia, and a serum ferritin level of 15 mg/L or less (12). IDA patients and HC had no intrinsic factor and anti-parietal cell autoantibodies. Normal white blood cell and reticulocyte counts were found in PA, IDA and HC. In eight patients (six females and two males, mean age 52; range 37-64 years) with PA and four HC (two females and two males, mean age 46; range: 42-48 years) following informed consent, biopsy specimens were obtained from the gastric mucosa in order to study LPMC.

**Table 1:** Sex (N, %) and age (mean ± SD; median and Q1-Q3) of study population.

Group	Sex	Age
**HC**	Female 21 (45%)	45.0 ± 14.1
(N = 47)	Male 26 (55%)	48.0 (34.2 - 57.0)
**IDA**	Female 17 (85%)	69.3 ± 22.7
(N = 20)	Male 3 (15%)	80.5 (44.5 - 85.7)
**PA**	Female 27 (63%)	69.5 ± 11.2
(N = 43)	Male 16 (37%)	72.0 (58.0 - 78.0)
**Total**	Female 65 (59%)	59.1 ± 19.5
**(N = 110)**	Male 45 (41%)	58.0 (45.0 - 77.0)

**Table 2:** Laboratory features of PA patients, IDA patients and controls.

Group	PCA pos	IFA pos	PCA & IFA pos
**HC**	0 (0%)	0 (0%)	0 (0%)
(N = 47)			
**IDA**	0 (0%)	0 (0%)	0 (0%)
(N = 20)			
**PA**	12/43 (28%)	15/43 (35%)	16/43 (37%)
(N = 43)			

PCA pos indicates patients with significantly detectable serum autoantibodies against gastric parietal cells. IFA pos indicates patients with significantly detectable serum autoantibodies against intrinsic factor. PCA & IFA pos indicates patients with both PCA and IFA positivity.

### Luminex Assay for IL-19, IL-20, IL-26, IL-28A and IL-29

Serum samples from 110 enrolled subjects were tested for IL-19, IL-20, IL-26, IL-28A and IL-29 levels with the Bio-Plex Pro™ assay (BIO-RAD, Hercules, California, USA) based on Luminex xMAP technology. Briefly, the 96 wells plate was seeded with fluorescently dyed magnetic beads, each with a specific color code to permit discrimination of tested analytes within multiplex suspension. Sera were diluted 1:4 with sample diluent provided with the kit. Calibrator vial was reconstituted according to the manufacturer’s instructions and the standard dilution series 1:3 was performed. Sera and calibrators were added following the plate layout. After 30 minutes incubation at room temperature (RT) and three washes, detection antibody was added and the plate was incubated 30 minutes further at RT. After streptavidin-phycoerythrin addition, the plate was finally washed and, after resuspended in assay buffer, data were acquired on MAGPIX System (Luminex, Texas, USA). Bio-Plex Manager™ software was used for data analysis of cytokines concentration by fitting fluorescence intensity values into the calibration curve.

### IL-19 Cytokine Production by Gastric Lamina Propria Mononuclear Cells

The gastric biopsies were obtained from eight PA patients and four healthy controls. LPMC were isolated by the DTT-EDTA-collagenase sequence as previously described (25). To induce cytokine production by gastric LPMC, 10^5^ cells were stimulated for 36 hours with phorbol 12-myristate 13-acetate (PMA) (10 ng/ml) plus ionomycin (0.5 mM) in 100 μl complete medium, as detailed elsewhere (26). Culture supernatants were assayed for IL-19 content. The quantitative determination of IL-19 was performed by Bio-Plex IL-19 singleplex assay (BIO-RAD).

### Statistical Analyses

Descriptive statistic was applied for the calculation of qualitative data, as well as for mean, standard deviation, median and interquartile range. Distributions of obtained results were evaluated for their normality and then compared by Student T-test or by Mann-Whitney U test.

*p*<0.05 was considered statistically significant.

Serum cytokine concentrations values falling below the lower limit of quantification (LLOQ) were replaced with one-half the respective LLOQ for descriptive purposes (27); no subject had values above the upper limit of quantification (ULOQ).

Luminex assay accuracy in discriminating between two compared groups was computed by ROC (Receiving Operating Characteristic) analysis. Plotting a ROC curve, the obtained AUC (Area Under the Curve) defines the test accuracy (28).

Statistical analysis were carried out using IBM^®^SPSS Statistic version 27.0.

## Results

### IL-19 Serum Levels Are Significantly Higher in Patients With Pernicious Anemia Than in Patients With Iron Deficiency Anemia and in Healthy Controls

**Figure 1:**
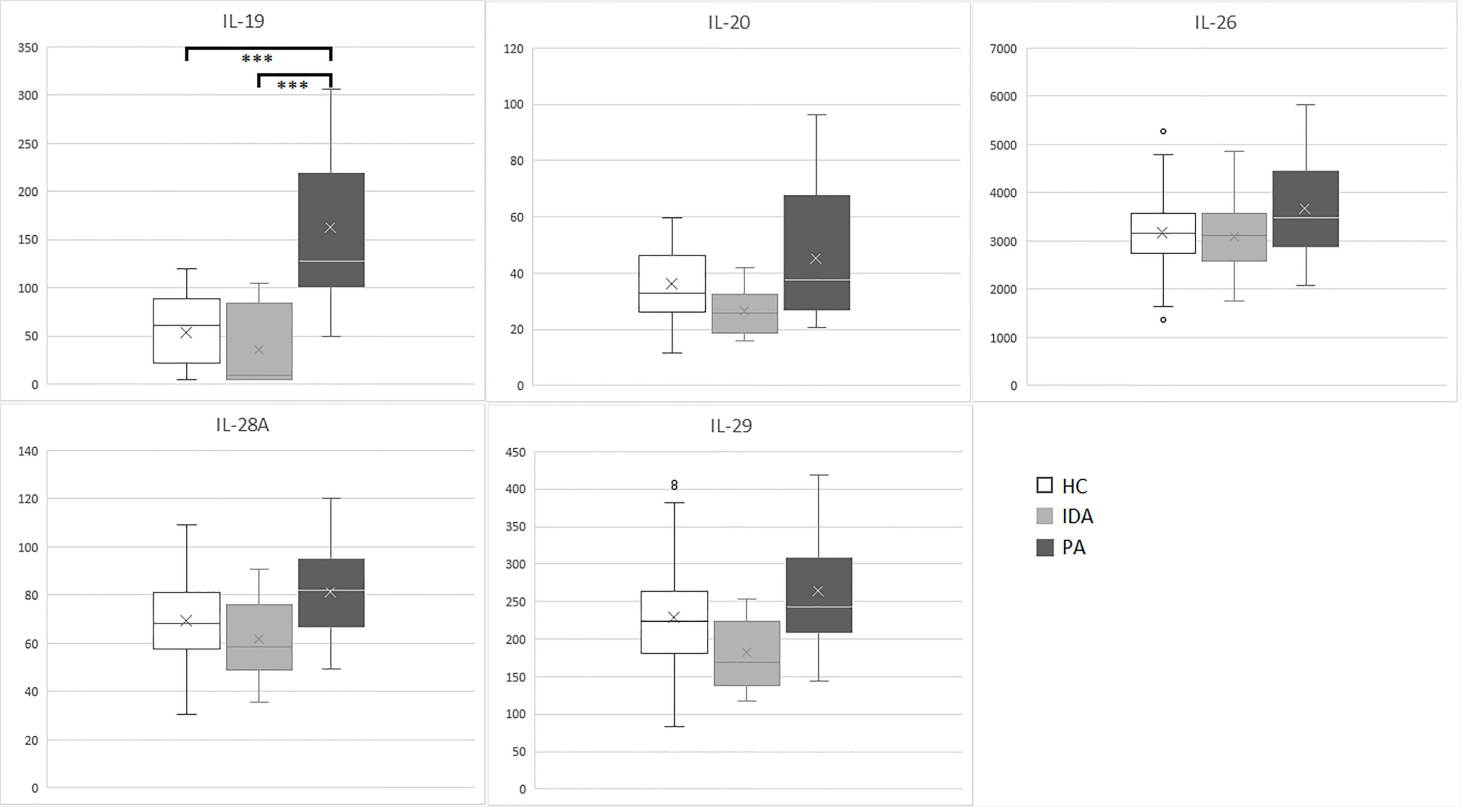
IL-19, IL-20, IL-26, IL-28A and IL-29 (pg/ml) levels in sera samples of enrolled subjects (PA, pernicious anemia; IDA, iron deficient anemia; HC, healthy control). ***p <0.001.

Results for the five cytokines in the groups of patients and controls are detailed in **Table 3**. Distribution of the levels of each cytokine is shown in **Figure 1**.

**Table 3:** Luminex assay results for IL-19, IL-20, IL-26, IL-28A and IL-29 levels in sera of HC, Healthy controls; IDA, Iron deficiency anemia patients; PA, Pernicious anemia patients.

Cytokine	LLOQ-ULOQ (pg/ml)		MEAN ± SD	*P*	*P*	*P*
				HC vs IDA	HC vs PA	IDA vs PA
**IL-19**	8.5 – 6223.8	**HC**	55.68 ± 36.75	0.096	**<0.001**	**<0.001**
		**IDA**	35.49 ± 40.97			
		**PA**	163.68 ± 75.96			
**IL-20**	2.7 – 5809.9	**HC**	36.08 ± 12.13	0.877	0.084	0.134
		**IDA**	26.52 ± 7.83			
		**PA**	45.44 ± 21.73			
**IL-26**	8.2 – 5969.7	**HC**	3154.28 ± 798.06	0.973	0.309	0.463
		**IDA**	3087.45 ± 747.33			
		**PA**	3699.23 ± 1056.83			
**IL-28A**	10.7 – 7813.5	**HC**	69.82 ± 17.36	0.914	0.622	0.627
		**IDA**	61.61 ± 16.20			
		**PA**	81.09 ± 18.85			
**IL-29**	5.2 – 11431.4	**HC**	230.23 ± 74.78	0.279	0.313	0.078
		**IDA**	181.87 ± 45.50			
		**PA**	266.69 ± 79.91			

Cytokines concentrations are expressed as pg/ml. The assay detection range is specified as lower-upper limit of quantification (LLOQ-ULOQ) for each tested analyte.

Significant differences were identified exclusively in IL-19 levels between patients with PA *vs*. HC (*p*<0.001) and between patients with PA *vs.* IDA patients (*p*<0.001). IL19 levels in PA, IDA and HC did not differ between males and females. No differences were found for the other cytokines.

### Serum Levels of IL-19 as a Useful Tool for the Discrimination Between Pernicious Anemia and Iron Deficient Anemia

**Figure 2:**
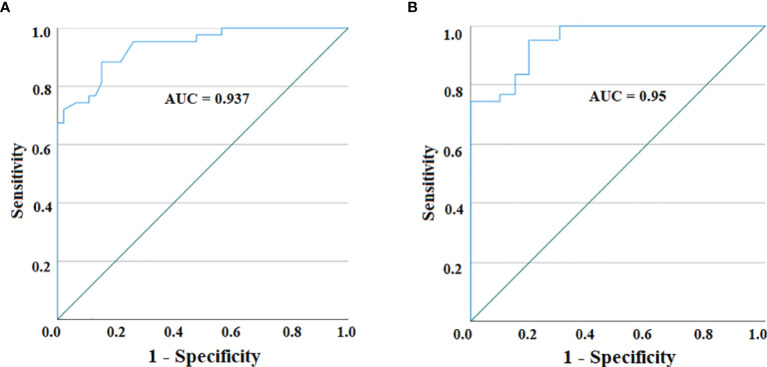
ROC curve for IL-19 serum levels in PA patients *vs*. HC **(A)** and between PA patients and IDA ones **(B)**. The AUC for both curves reveal a highly accurate test performance (0.9 ≤ AUC < 1).

With the purpose of using serum IL-19 quantification by Luminex assay as a useful tool to discriminate PA from IDA, we calculated the AUC of the ROC curve to assess the test performance. As graphed in **Figure 2A**, the ROC curve analyzing serum IL-19 amounts in HC compared to PA patients, at a cutoff of 91.05 pg/ml, provides an AUC = 0.937, with a sensitivity of 88.4% and a specificity of 84.8%. The comparison by ROC analysis of the IL-19 serum amounts in PA and IDA patients, found an AUC = 0.95, with a sensitivity of 95.3% and a specificity of 80.0%, choosing as best cutoff 88.08 pg/ml (**Figure 2B**).

### IL-19 Production by Lamina Propria Mononuclear Cells Derived From the Gastric Mucosa of Patients With Pernicious Anemia

**Figure 3:**
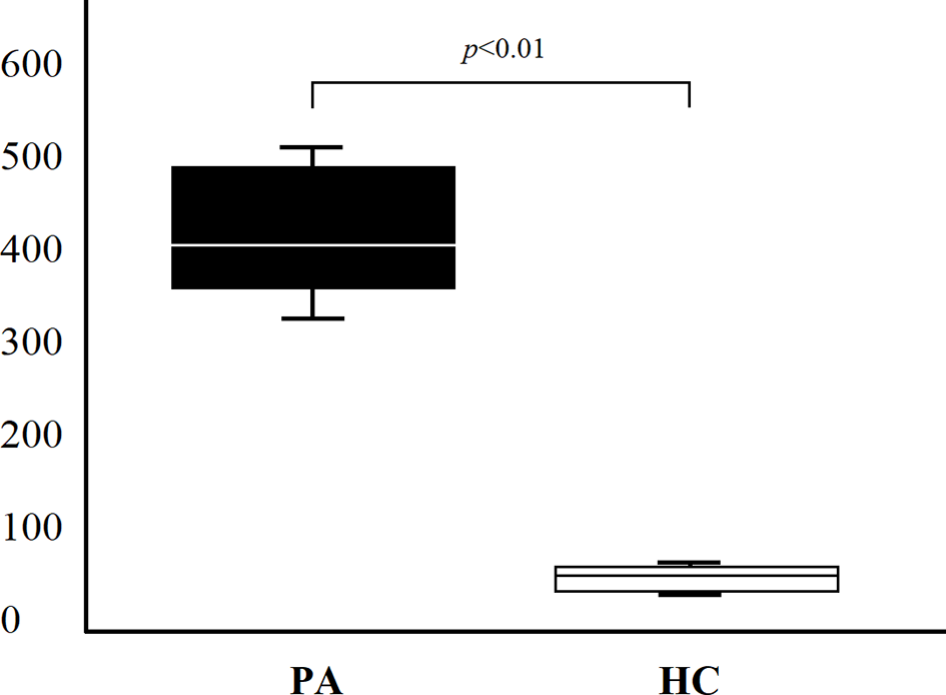
IL-19 production (pg/ml) of lamina propria mononuclear cells obtained from gastric specimens of 8 PA patients and 4 HC. Comparison of gastric IL-19 (mean ± SD) production between PA patients and HC.

On the basis of results obtained at serum level, we then asked whether IL-19 is produced *in vivo* by patients with PA and autoimmune gastritis. Since gastric mucosal involvement was almost invariably present in patients with PA, gastric specimens were considered an appropriate source of *in vivo* LPMC. Eight patients with PA and four healthy controls studied at serum level were investigated for IL-19 cytokine production by LPMC derived from their gastric mucosa. Gastric LPMC were isolated by the DTT-EDTA-collagenase sequence. Following stimulation with PMA and ionomycin we measured IL-19 production by gastric LPMC in supernatants. A statistically significant difference of IL-19 production (*p* = 0.0045) was found between supernatant of gastric LPMC derived from all PA patients (mean 420.67 ± 68.14 pg/ml) and HC ones (mean 53.69 ± 10.92 pg/ml), as shown in **Figure 3**.

## Discussion

The present study revealed several major findings that are relevant to the management and understanding the immunological basis of pernicious anemia and autoimmune gastritis.

We investigated the serum levels of different cytokines, such as IL-19, IL-20, IL-26, IL-28A and IL-29 in patients with pernicious anemia and autoimmune gastritis or iron deficient anemia without autoimmune gastritis. No differences in IL-20, IL-26, IL-28A and IL-29 serum levels were present among untreated PA patients, IDA patients or HC. On the other hand, significantly higher levels of IL-19 were found in the sera of PA patients compared to IDA or HC. It is of note that PA is a disease with a clear autoimmune pathogenesis, whose immunopathological features are gastric autoreactive T cells and autoreactive B cells specific for proton pump ATPase and IF, whereas the patients with IDA studied in this research had no autoimmune disease (29, 30). Our results support the notion that IL-19 is a relevant cytokine for the immunopathogenesis of PA but not of IDA.

We can hypothesize that the high serum level of IL-19 found in PA patients might be inversely related to the serum levels of vitamin B_12_ and that the substitution therapy with B_12_ will lead to both normal serum levels of B_12_ and IL-19. It can be further speculated that therapeutic regimen able to reduce IL-19 levels, such as monoclonal antibodies or siRNA, might be useful for the treatment of PA. Given that this investigation represents a pilot single-center study, future multi-center studies dealing with serum IL-19 levels would be very useful for a better definition of the test in PA and IDA.

To investigate whether at gastric level there was any IL-19 production *in vivo*, we analyzed the IL-19 cytokine production of LPMC derived from the gastric mucosa of patients with PA and HC. We found that LPMC obtained from all PA patients, but not HC, were able to produce high levels of IL-19. It was recently reported in patients with PA that gastric T cells specific for intrinsic factor produced very high level of IL-17 and TNF-α (8). It is of high interest that IL-19 expression can be induced by IL-17, TNF-α and lipopolysaccharide. Moreover, epithelial cells produce IL-19 following stimulation with IL-17, IL-22 and IL-1β (20). In PA patients a different IL-19 cytokine production might be related not only to the local inflammation but also to a different bone stimulation or to other yet unknown interfering factors in IL-19 cytokine production.

Altogether, the results obtained so far indicate that IL-19 serum levels are significantly increased in patients with PA and that IL-19 is produced *in vivo* in the stomach of PA patients. Thus, we suggest that serum levels of IL-19 may represent a novel important tool to discriminate PA with autoimmune gastritis from IDA without autoimmune gastritis and that the measurement of serum IL-19 might be useful for monitoring the response to B_12_ vitamin therapy in PA patients.

## Data Availability Statement

The raw data supporting the conclusions of this article will be made available by the authors, without undue reservation.

## Ethics Statement

The studies involving human participants were reviewed and approved by Ethical Committee Area Vasta Centro Firenze. The patients/participants provided their written informed consent to participate in this study.

## Author Contributions

CDB, AAn, NB and MMD'E conceived and designed the study with MMD'E supervision. CDB, MPP, NC, MB, LP, AAz, SP, SD'E, FC, DO-P performed the experiments; analysis and interpretation of data were conducted by CDB, AAn, NB, MMD'E. CDB, AAn, NB, MMD'E wrote, reviewed and edited the manuscript. All the authors contributed to the article and approved the submitted version.

## Funding

This work was supported by the Italian Ministry of University (grant 2010P3S8BR_005).

## Conflict of Interest

The authors declare that the research was conducted in the absence of any commercial or financial relationships that could be construed as a potential conflict of interest.

## Publisher’s Note

All claims expressed in this article are solely those of the authors and do not necessarily represent those of their affiliated organizations, or those of the publisher, the editors and the reviewers. Any product that may be evaluated in this article, or claim that may be made by its manufacturer, is not guaranteed or endorsed by the publisher.
